# Unveiling a sustainable approach to cancer treatment: the antitumor activity of amygdalin and cell-free supernatant of *Lacticaseibacillus rhamnosus*

**DOI:** 10.1007/s12223-025-01289-x

**Published:** 2025-07-10

**Authors:** Omnia Karem M. Riad, Hagar F. Forsan, Basma Abdulsamad, Heba Mohammed Refat M. Selim, Wafaa S. Khalaf

**Affiliations:** 1 Department of Microbiology and Immunology, Faculty of Pharmacy (Girls), Al-Azhar University, Nasr City, Cairo, 11751 Egypt; 2 Dairy Chemistry Research Department, Animal Production Research Institute (APRI), Agricultural Research Center (ARC), Dokki, Giza, Egypt; 3 Immunology and Allergy Department, Medical Research Institute, Alexandria University, Alexandria, Egypt; 4 Department of Pharmaceutical Sciences, College of Pharmacy, AlMaarefa University, P.O. Box 71666, 11597 Riyadh, Saudi Arabia

**Keywords:** Amygdalin, Cancer treatment, Cell-free supernatant of *Lacticaseibacillus rhamnosus*, Environmental sustainability

## Abstract

The pharmaceutical industry increasingly prioritizes environmental sustainability across its strategies, focusing on developing eco-friendly anti-cancer treatments. This is particularly important considering many anti-cancer drugs are not fully metabolized and pollute aquatic ecosystems. Amygdalin, a plant-derived compound, and cell-free supernatants (CFS) of *Lacticaseibacillus rhamnosus* show promise as sustainable anti-cancer agents. This study evaluated the potential anti-cancer activity of amygdalin and *L. rhamnosus* CFS against MCF-7 and A549 cancer cell lines. Cell viability was assessed using the MTT assay in response to monotherapy and combination therapy treatments. The induction of apoptosis was investigated using flow cytometric analyses. The combination demonstrated a synergistic effect on MCF-7 and A549 cell lines, with a combination index (CI) value of 0.8159 ± 0.0245 and 0.6422 ± 0.0316, respectively. Further analysis using propidium iodide (PI) staining and Annexin V confirmed increased apoptosis in combination-treated cells compared to those treated with amygdalin alone. These findings suggest that the combination of amygdalin and CFS of *L. rhamnosus* exhibits a synergistic anti-cancer effect against MCF-7 and A549 cells. Therefore, this combination is considered sustainable, eco-friendly, and effective anti-cancer treatment. This is the first study to investigate the anti-cancer effect of amygdalin in combination with *L. rhamnosus* cell-free supernatant (CFS) while considering its potential for environmental sustainability. Further research in a xenograft animal model is warranted to validate these findings.

## Introduction

Despite advancements in cancer treatment, the disease remains a leading cause of mortality. Tolerance and adherence are further hampered by the toxicity of traditional medicines. Furthermore, both classical and new cancer treatment techniques (chemotherapy, surgery, radiation, immunotherapy, and targeted therapy) have adverse effects that negatively affect the quality of human life (Mun et al. 2018; Khalaf et al. 2019). Anti-cancer medicines are often poorly digested, and patients expel them unaltered into their urine and feces (Turci et al. 2003; Johnson et al. 2008; Mohammed et al. 2024). This means that these drugs leak into the aquatic environment, as several anti-cancer medications are frequently not eliminated by wastewater treatment plants, posing a threat to aquatic microbial communities and, consequently, being harmful to humans (Li et al. 2021).

Another neglected issue in producing new drugs is the conservation of environmental resources. Integrating sustainability principles into the pharmaceutical industry’s microbial processes and product development presents significant challenges and has slowly been gaining popularity. As a result, the search for new sustainable, effective, and eco-friendly anti-cancer treatments is necessary (Mun et al. 2018; Wynendaele et al. 2021).

Amygdalin is one of the promising plant-derived compounds with claimed anti-cancer activity. Amygdalin, a cyanogenic glycoside, is a naturally occurring compound found in almonds and other Rosaceae plant seeds, primarily in apricot and almond kernels (He et al. 2020). Amygdalin, a cyanogenic diglucoside, has grown in popularity among cancer patients as a supplement to or replacement for traditional therapies. Nonetheless, evidence-based research is scarce on amygdalin, and its benefits are debatable (Blaheta et al. 2016). In vitro studies have demonstrated the anti-proliferative effects of amygdalin on various cancer cell lines (Sireesha et al. 2019). However, statements published by the FDA in the late 1970 s on a patient study (Rosen 1979) contradicted this. Since then, numerous studies have been published proving both the medicinal, particularly anti-cancer, benefits of amygdalin and the toxicity associated with excessive ingestion of bitter almond-containing amygdalin (Jaszczak-Wilke et al. 2021). Several studies have stated that amygdalin has anti-cancer, anti-inflammatory, anti-atherosclerotic, and antifibrotic activity. It also has immunomodulatory and analgesic properties and can improve cardiac hypertrophy, neurodegeneration, and blood glucose levels (He et al. 2020). Furthermore, research indicates that the toxicity of amygdalin arises primarily from the release of benzaldehyde and hydrogen cyanide upon oral ingestion. Intravenous administration exhibits significantly lower toxicity compared to oral ingestion. Furthermore, a daily oral dose of 0.6 to 1 g appears well tolerated (He et al. 2020).

In the current study, we investigated amygdalin for its potential anti-cancer activity, given its common use as a complementary and alternative medicine (CAM) among patients and healers. The selection of amygdalin was motivated by the well-documented dissatisfaction of patients with conventional chemotherapeutics, leading a high percentage (50–90%) of cancer patients to seek CAM options, as reported in Polish, French, Swedish, and German studies (Renet et al. 2021; Jędrzejewska et al. 2021; Källman et al. 2023; Stöcker et al. 2023). Notably, a German survey conducted among physicians and healers found that 66% of respondents purchased amygdalin from pharmacies, with many encouraging its use. Clinical data revealed that intravenous amygdalin administration achieved 90% disease progression prevention and 55% symptom relief (Markowitsch et al. 2024). Additionally, recent studies have demonstrated amygdalin’s potent antibacterial activity against multidrug-resistant *S. aureus*, a particularly relevant finding given the high susceptibility of malignancy patients to such infections (Wang et al. 2024).

Given the heightened susceptibility of cancer patients to infections, we combined amygdalin with a natural, sustainable compound possessing both established antibacterial properties and emerging evidence of anti-cancer activity—specifically, cell-free supernatants (CFS) from *lactic acid bacteria* (LAB). This strategic combination aims to enhance therapeutic efficacy while potentially reducing the required amygdalin dose and its associated toxicity. Supporting this approach, in vitro studies demonstrate that LAB exerts anti-proliferative effects on multiple human cancer cell lines (Abedin-Do et al. 2015). Their influence on metabolic, immunologic, and defensive processes further contributes to delaying carcinogenesis. For instance, metabolites secreted by *Lactobacillus plantarum* YYC-3 inhibit colon cancer cell dissemination by suppressing the VEGF-MMP2/9 signaling pathway (Yue et al. 2020).

Research has demonstrated that *L. delbrueckii* subsp. lactis suppresses gynecological cancer progression by inhibiting SiHa cell proliferation and inducing apoptosis (Bi et al. 2023). Similarly, *Lactiplantibacillus plantarum* exerts selective cytotoxicity against KB and OSCC cancer cell lines, triggering apoptosis without significant harm to healthy cells (Nami et al. 2024; Wonglapsuwan et al. 2024).

Due to their selective cytotoxicity against cancer cells and minimal impact on normal cells, *Lactobacillus spp.* are promising nutraceutical agents for cancer treatment (Nami et al. 2024). This study investigates the combined use of two sustainable green compounds: amygdalin and cell-free supernatant (CFS) from *Lacticaseibacillus rhamnosus* (formerly *L. rhamnosus* ATCC 7469), which exhibit diverse biological activities (Wonglapsuwan et al. 2024). We evaluated their anti-cancer effects on MCF-7 and A549 cell lines, quantifying cell viability via flow cytometry. Additionally, we optimized the combination of IC50 doses of both compounds to maximize anti-cancer efficacy at minimal concentrations.

## Materials and methods

### Amygdalin (vitamin B17)

99.5% Organic amygdalin vegetarian (100% Apricot Kernel Extract) was purchased as 100 mg capsule from Wellness Extract, USA.

### Bacterial cultures

*Lacticaseibacillus rhamnosus* (ATCC 7469) was bought as an active culture from ASUCairo MIRCEN, Ain Sham University, Egypt.* L. rhamnosus* (ATCC 7469) was cultured in MRS broth (Oxoid, England). It was preserved in MRS broth with 20% glycerol (V/V) at − 20 °C.

### Cell lines and culture condition

Human breast cancer cells (MCF-7) and human alveolar basal epithelial adenocarcinoma cells (A549) were procured from the American Type Culture Collection (ATCC, Manassas, VA, USA). Cells were cultured in Dulbecco’s Modified Eagle Medium (DMEM) supplemented with 10% (v/v) heat-inactivated fetal bovine serum (FBS), 100 U/mL penicillin, and 100 μg/mL streptomycin (Gibco, Billings, MT, USA). All cell lines were maintained at 37 °C in a humidified incubator with 5% CO₂ and 95% air.

### Preparation of a CFS

*Lacticaseibacillus rhamnosus* (ATCC 7469) was cultured aerobically at 37 °C in de Man, Rogosa, and Sharpe (MRS) broth (Oxoid, UK) for 24 h. Bacterial cells were removed by centrifugation at 10,000 g for 10 min at 4 °C. The supernatant was sterile-filtered through a 0.22-μm pore-size membrane (Millipore, Bedford, MA, USA). For cell treatment, lyophilized CFS was reconstituted in RPMI-1640 medium or dimethyl sulfoxide (DMSO), followed by re-sterilization using a 0.22-μm filter.

### Cytotoxicity assay (MTT assay)

The MTT assay is extensively used to assess the cytotoxicity of treatments to cells (Ghasemi et al. 2023). To form a confluent monolayer, 1 × 10^5^ cells per mL (100 µL per well) of either MCF-7 or A549 cells were seeded in a 96-well tissue culture plate and incubated at 37 °C for 24 h. The cells were then washed twice with PBS. Serial dilutions of amygdalin, CFS, and their combinations (covering concentrations including IC₅₀ values) were prepared in RPMI-1640 medium containing 2% FBS. A volume of 100 µL of each dilution was added to the wells in triplicate, with the control wells receiving medium only. The plate was incubated at 5% CO₂ at 37 °C. Cell morphology was monitored for signs of toxicity (e.g., cell detachment, rounding, or granulation). After incubation, 20 µL of MTT solution (5 mg/mL in PBS; Bio Basics Canada Inc.) was added to each well, followed by incubation at 37 °C for 1–5 h to allow MTT metabolism. The medium containing formazan crystals was removed, and 200 µL of DMSO was added to each well to dissolve the crystals. The plate was shaken for 5 min at 150 rpm to ensure complete solubilization. Absorbance was measured at 560 nm using a microplate reader, with background subtraction at 620 nm. In MTT assay, the negative control consisted of cells cultured under standard conditions without any treatment with amygdalin and CFS of *L. rhamnosus* therapy. These cells were maintained in complete growth medium and incubated for the same duration as the treatment groups. The purpose of this control was to establish the baseline metabolic activity and viability of the untreated cells, which serves as a reference point for evaluating the cytotoxic effects of amygdalin.

### Apoptosis assay

Flow cytometry-based assay is commonly used to identify apoptotic cells uses staining with fluorochrome-conjugated annexin-V (Lieschke et al. 2022). Accordingly, cell apoptosis was measured using flow cytometric analysis, utilizing the FITC Annexin V Apoptosis Detection Kit (BD Biosciences). Following the manufacturer’s protocol, apoptosis was induced in cells by treatment with a concentration of the test compound equal to its IC50 value, as determined through the MTT assay. For MCF-7 cells, treatments included 55.93 μg/mL amygdalin, 354 μg/mL CFS of *L. rhamnosus*, and combination of both (24.86 μg/mL amygdalin + 131.5 μg/mL CFS). For A549 cells, treatments included 54.73 μg/mL amygdalin, 293 μg/mL CFS of *L. rhamnosus*, and combination of both (17.95 μg/mL amygdalin + 92.09 μg/mL CFS), which were added and incubated for 48 h. A total of 1–5 × 10^5^ cells were harvested via centrifugation and resuspended in binding buffer (500 μL, 1 ×). Trypsin–EDTA was used to detach adherent cells, then washed with DMEM media. Subsequently, the cells were stained with Annexin V-FITC (5 µL) and propidium iodide (PI) μL of 50 μg/mL, for 5 min in the dark at 25 °C. Finally, cellular apoptosis was quantified using a BD FACS Calibur flow cytometer.

Cell apoptosis was analyzed by flow cytometry using the FITC Annexin V Apoptosis Detection Kit (BD Biosciences). Following the manufacturer’s protocol, apoptosis was induced by treating cells with test compounds at their IC50 values (determined by the MTT assay).

All treatments lasted 48 h. Cells (1–5 × 10^5^) were harvested by centrifugation and resuspended in 500 μL of 1 × binding buffer. Adherent cells were detached using trypsin–EDTA and washed with DMEM. Cells were then stained with 5 μL Annexin V-FITC and 5 μL propidium iodide (PI, 50 μg/mL) for 5 min at 25 °C in the dark. Apoptosis was quantified using a BD FACS Calibur flow cytometer. The negative control was established using MCF-7 or A549 healthy, untreated cells. These cells had been cultured under the same conditions to ensure high viability and to minimize any background apoptosis.

### Statistical analysis

Dose–response curves were generated through non-linear regression analysis using GraphPad Prism 5.02 software. Cell viability data, normalized to untreated controls, were plotted against logarithmically transformed drug concentrations. Results are expressed as mean ± standard error (SE). Statistical significance (*p* < 0.01) was determined by two-way ANOVA followed by Bonferroni’s post-hoc test. The combination index (CI) values for amygdalin and *L. rhamnosus* CFS against A549 and MCF-7 cell lines were calculated according to the Chou-Talalay method (Chou 2010).

## Results

Cytotoxicity of amygdalin and CFS of *Lacticaseibacillus rhamnosus* on MCF-7 and A549 cell lines.

**Fig. 1:**
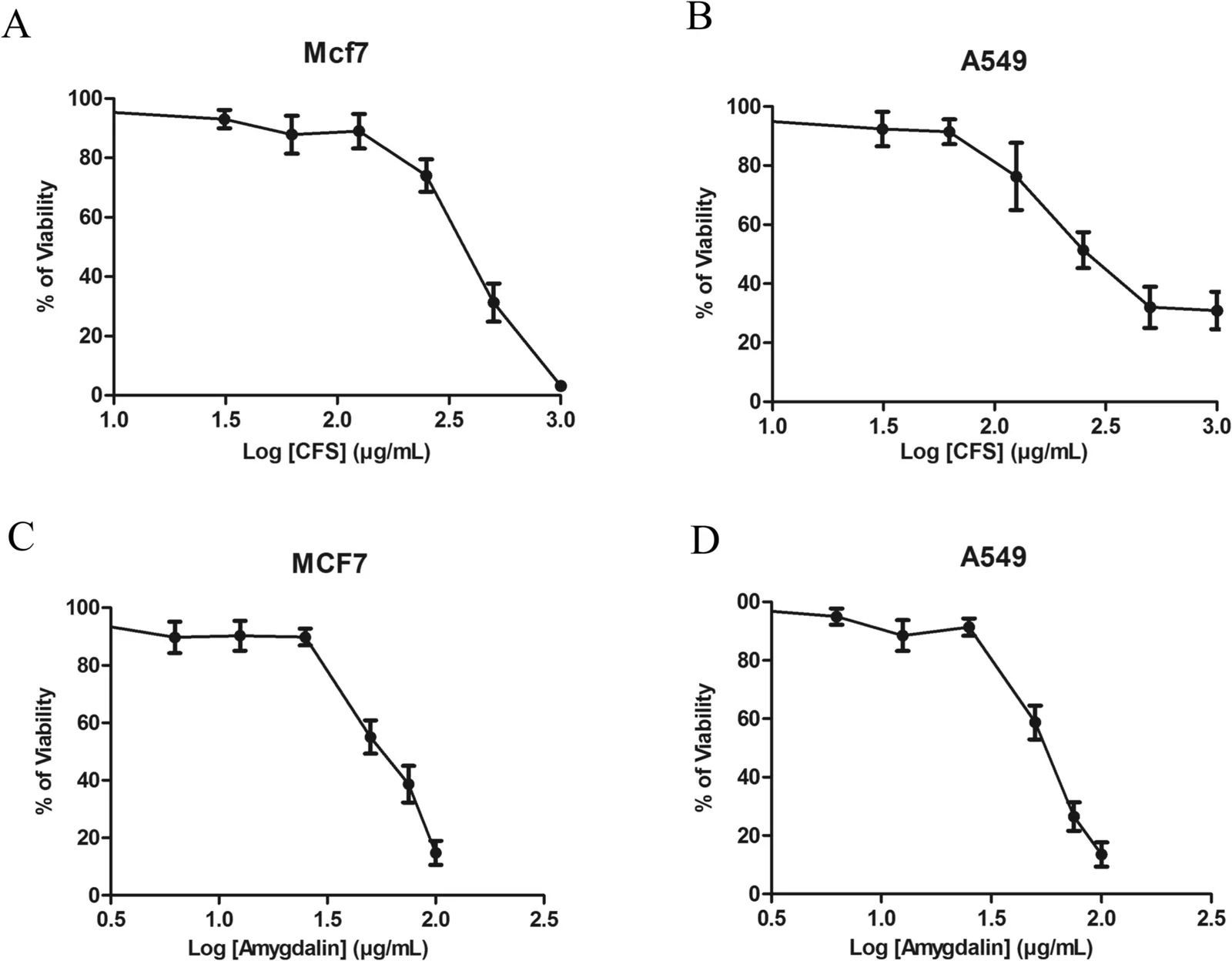
Dose–response curves. MCF-7 and A549 cell lines were treated with *L. rhamnosus* cell-free supernatant (**A** and **B**) or amygdalin (**C** and **D**) at varying concentrations. Cell viability was assessed using the resazurin assay, with data presented as mean ± standard error (SE)

The MTT assay was used to calculate the IC50 value of each compound individually, before examining the effect of amygdalin combined with CFS of *L. rhamnosus*. The incubation was done at various doses, and dose–response curves were constructed for each drug on two different cell lines (Fig. 1). Data obtained from the MTT assay were normalized to the absorbance values of the negative control, which was set at 100% viability, allowing for the calculation of relative cell viability percentages in the treated groups. Both amygdalin and CFS of *L. rhamnosus* therapy inhibited MCF-7 and A549 cell growth in a dose-dependent mode (Fig. 1).

The IC50 of amygdalin and CFS of *L. rhamnosus* on the MCF-7 cell line, were 55.93 ± 2.16 and 354 ± 5.05 µg/mL, respectively. While the effect of amygdalin and CFS of *L. rhamnosus* on the A549 cell line was 54.73 ± 1.67 and 293 ± 3.09 µg/mL, respectively (Table 1).

**Table 1:** Cytotoxicity of amygdalin and CFS of *L. rhamnosus* on MCF-7 and A549 cell lines. IC50 values are given as mean ± SD

	MCF-7		A549	
	Monotherapy (μg/mL)	Combination therapy (μg/mL)	Monotherapy (μg/mL)	Combination therapy (μg/mL)
Amygdalin	55.93 ± 2.16	24.86 ± 0.185	54.73 ± 1.67	17.95 ± 0.484
CFS of *L. rhamnosus*	354 ± 5.05	131.5 ± 0.568	293 ± 3.09	92.09 ± 0.821

**Fig. 2:**
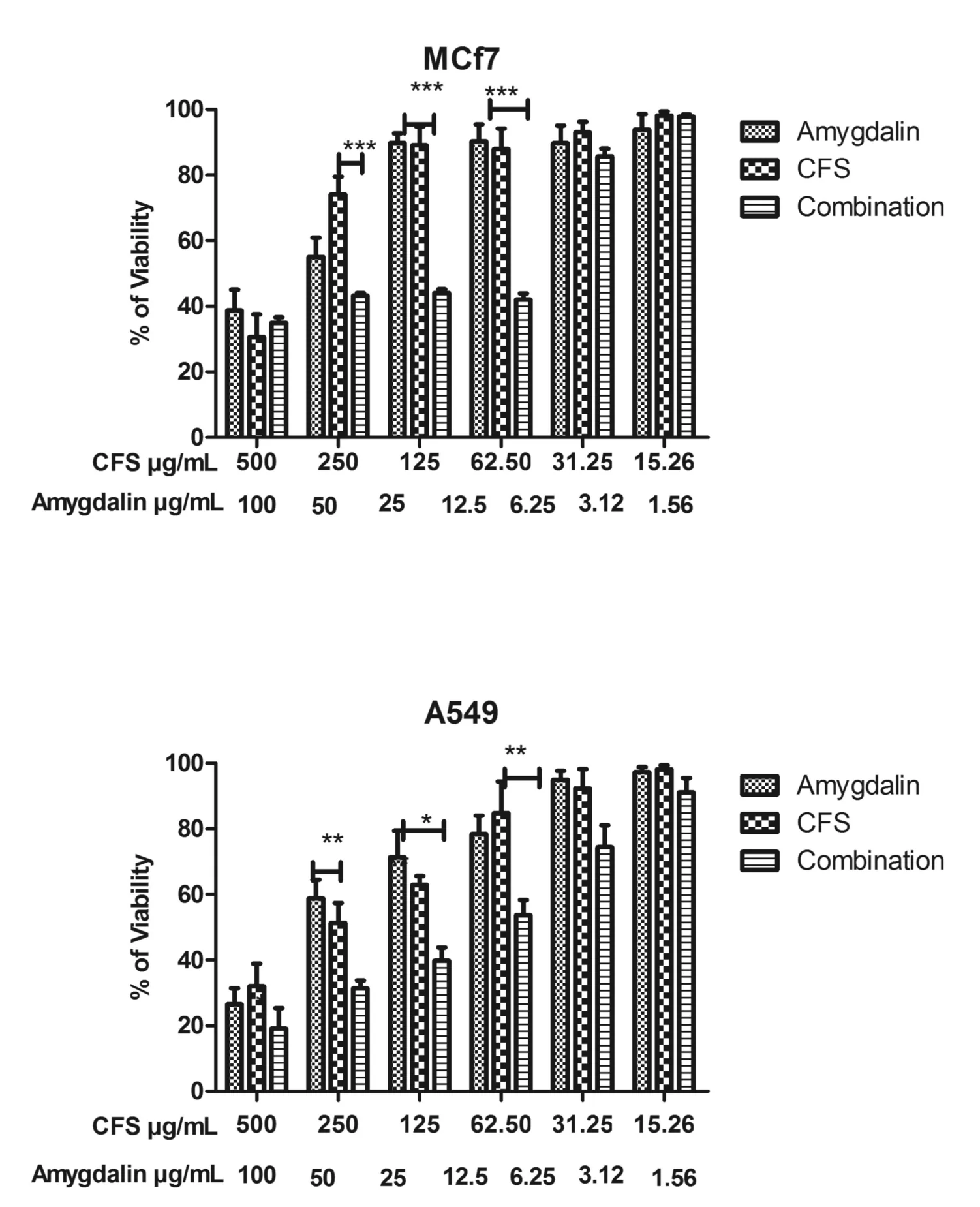
Anti-proliferative effects of *L. rhamnosus* cell-free supernatant (CFS) and amygdalin as single agents and in combination on A549 and MCF-7 cell lines. Data are presented as mean ± standard error (SE). *p < 0.01 and **p < 0.001 indicate statistical significance

To evaluate the combinatorial effect, a fixed ratio of amygdalin and *L. rhamnosus* CFS of 1:10 was employed. This ratio was based on the IC50 values determined for each agent when used as monotherapy. The combination regimen included concentrations both at, above, and below the IC50 values. Figure 2 showed enhanced anti-proliferative activity of amygdalin and *L. rhamnosus* CFS combination in MCF-7 cells. The combination of amygdalin and CFS exhibited significantly greater anti-proliferative effects against MCF-7 cells compared to either agent alone. This combination resulted in a decrease in the IC50 values for amygdalin to 24.86 ± 0.185 µg/mL and for CFS to 131.5 ± 0.568 µg/mL, respectively (Table 1).

In A549 lung cancer cells, amygdalin and *L. rhamnosus* CFS were evaluated as single agents and in combination. The IC50 values for amygdalin and CFS as monotherapies were determined to be 54.73 ± 1.67 µg/mL and 293 ± 3.09 µg/mL, respectively. Upon combination, a significant reduction in IC50 values was observed for both agents, with values decreasing to 17.95 ± 0.484 µg/mL for amygdalin and 92.09 ± 0.821 µg/mL for CFS (Table 1). Consistent with these findings, Fig. 2 demonstrates that the combination of amygdalin and CFS significantly inhibited the growth of both MCF-7 and A549 cell lines and lowered the IC50 of amygdalin and CFS of *L. rhamnosus*.

### Synergistic effect of combination

The combination index (CI) values for the drug combination against MCF-7 and A549 cell lines were determined using the Chou-Talalay method. The calculated CI values for MCF-7 and A549 cell lines were 0.8159 ± 2.45 and 0.6422 ± 3.16, respectively.

### Apoptotic activity of amygdalin and CFS of *Lacticaseibacillus rhamnosus*

The apoptotic activity of amygdalin and CFS of *L. rhamnosus* is summarized in Table 2. CFS of *Lactobacillus* induced apoptosis in 26.18% and 19.51% of cells in A549 and MCF-7 cell lines, respectively. In contrast, amygdalin alone induced late apoptosis and necrosis with a higher percentage than apoptosis. Finally, the combination of both amygdalin and CFS of *L. rhamnosus* induced apoptosis in 23.28% and 19.22% of cells in A549 and MCF-7 cell lines, respectively (Table 2).

**Table 2:** Apoptotic activity of amygdalin and CFS of *L. rhamnosus* on MCF-7 and A549 cell lines

Sample	Apoptosis %		Necrosis %
	**Early**	**Late**	
CFS/A549	26.18	8.59	3.82
CFS/MCF-7	19.51	11.47	1.63
Amygdalin/A549	2.59	7.97	4.32
Amygdalin/MCF-7	1.81	11.94	9.81
Combination/A549	23.28	4.32	1.94
Combination/MCF-7	19.22	12.08	4.43
Control A549	0.44	0.26	1.34
Control MCF-7	0.39	0.17	1.17

## Discussion

While the current anti-cancer drugs are the best available drugs for treating malignancies, they may be an indirect cause of cancer by polluting the environment due to the difficulty of their decomposition. They are not fully metabolized and excreted in patients’ stools and leak into groundwater and the environment, representing a hazard to the ecosystem (Johnson et al. 2008; Li et al. 2021; Turci et al. 2003).

Safe disposal of antineoplastic agents increases the economic burden, requiring incineration at a high temperature above 1200 °C, immobilization (waste encapsulation), or monetization (WHO 1999). Many developing countries cannot withstand these expensive methods, and they have become the main cause of environmental contamination. Therefore, more attention should be paid to sustainability, especially in the pharmaceutical field and in developing new anti-cancer agents that are green, sustainable, and eco-friendly chemotherapeutics. This can be achieved by increasing awareness of the importance of sustainability among research institutions, relevant authorities, and industrial organizations (Wynendaele et al. 2021). From this point of view, this study aimed to test the efficacy of natural products in treating cancer cells by using green, sustainable compounds such as amygdalin and CFS from *L. rhamnosus*, which are plant-derived and bacteria-derived compounds.

Although ecological information about amygdalin is still lacking, the available data confirm that at concentrations of 0.1% or greater, amygdalin includes no components that are persistent, bioaccumulative, and toxic (PBT) or very persistent and very bioaccumulative (vPvB) (Sigma-Aldrich 2025). Although amygdalin is an organocyanide compound, it does not release its cyanide content in wastewater, whether chlorinated or not (Yi et al. 2002). Recent research has demonstrated that amygdalin exhibits anti-cancer and apoptotic effects against breast cancer cells by inhibiting cellular proteolytic pathways (Cecarini et al. 2022). Amygdalin triggers apoptosis by activating BAX and caspase-3 while downregulating PARP-1 (Saleem et al. 2019; Al-Khafaji and Taskin Tok 2021). Moreover, it reduces the percentage of cells that enter the S and G2/M phases by causing cell cycle arrest at the G0/G1 transition. As a result, limiting cell proliferation and growth is advocated to speed up the slowdown of the cell cycle (Saleem et al. 2019).

However, amygdalin is a cyanogenic glycoside found in certain seeds, such as bitter almonds, and undergoes enzymatic hydrolysis by β-glucosidase, leading to the release of hydrogen cyanide (HCN) (Fernandes and Billa 2025). The production of HCN poses serious health risks, including toxicity and poisoning, necessitating strict control measures to prevent adverse effects (Kadekar et al. 2024; Huang et al. 2025). Cyanide levels in the body are influenced by several factors, including the gut microbiota composition (Tokpohozin et al. 2016). Notably, *Bacteroides* exhibit high glucosidase activity, leading to the release of cyanide during the symbiotic digestion of amygdalin. Studies by Zhang et al. (2014) have shown that bacteria from the Bacteroidetes phylum dominate the gut microbiota (Zhang et al. 2014). These bacteria possess a high abundance of the glucosidase gene (Blaheta et al. 2016). Bacteroidetes, a common colon resident, has been found to produce the enzyme β-glucosidase. Anaerobic gut bacteria ferment pyruvate to generate lactic acid (Padayatty et al. 2010). This reduction in pH enhances glucosidase activity, which can be detrimental, as it facilitates the hydrolysis of amygdalin into cyanide, contributing to its toxic effects. β-glucosidases in the colon are directly involved in amygdalin hydrolysis (Blaheta et al. 2016).

Additionally, research findings confirm that *Lactobacillus* strains in the human gut possess both α- and β-glucosidase inhibitory activities (Panwar et al. 2014). Animal studies indicated that supplementation with *Lactobacillus* and its CFS reduces β-glucuronidase activity, suggesting a decline in β-glucuronidase-encoding Bacteroidetes populations (Baffoni et al. 2012; Hullar et al. 2014; Duda-Chodak et al. 2015) and hence the decrease in amygdalin toxicity. Cyanide produced during amygdalin hydrolysis in low doses is utilized in complementary and alternative medicine (CAM) due to its ability to bind cytochrome oxidase c and a3, thereby inhibiting cellular respiration and DNA synthesis through reactive oxygen species generation. This process ultimately blocks nutrient uptake and induces cell lysis (Bromley et al. 2005; Kuugbee et al. 2016). However, findings by Blaheta et al. (2016) suggest that cyanide is not the primary factor responsible for amygdalin’s antitumor properties, as its effects persist even in the absence of β-glucosidase (Blaheta et al. 2016; Kadekar et al. 2024).

The *L. rhamnosus* bacterium has been deemed eligible for a qualified presumption of safety (QPS) evaluation by the European Food Safety Authority (EFSA). This indicates that using this strain as a silage supplement is safe for consumers, animals, and the environment. The strain’s confirmed identity and the lack of any acquired antimicrobial resistance determinants of concern serve as the foundation for this evaluation (EFSA 2021). The standard strain *L. rhamnosus* ATCC 7469 is used in this study due to its fame for food production and agricultural research. The American Type Culture Collection (ATCC) states that it is only intended to be used in laboratory research and not as a medication, for human or animal consumption, or for diagnostic purposes (ATCC n.d.). Several studies have published research about the different biological activities of *L. rhamnosus* ATCC 7469 as an antibacterial, antifungal, antitoxin, or immune modulator (Jorjão et al. 2015; Abu-Elfotuh et al. 2023; Abdel-Nasser et al. 2023; Salem-Bekhit et al. 2023; Zaghloul et al. 2023; American Type Culture Collection (ATCC) (2025) *Lacticaseibacillus rhamnosus* (ATCC® 7469TM)).

Therefore, the combination of amygdalin and CFS of *Lactobacillus* is considered safe and offers protection against cyanide toxicity resulting from amygdalin metabolism. Accordingly, the current study examined the antitumor activity of amygdalin and cell-free supernatant of *L. rhamnosus* alone and in combination. Breast cancer and lung cancer are the most common and leading causes of death worldwide (El-Toukhy et al. 2023; Jokhadze et al. 2024; Karim et al. 2025). Therefore, the current study selected MCF-7 (human breast cancer cell line) and A549 (human lung cancer cell line) to test the anti-cancer activity of both amygdalin and CFS of *L. rhamnosus*. These two cell lines have been used in many studies to test the efficacy of new compounds as anti-cancer agents, e.g., studies by Jeong et al. (2024) and Görmez et al. (2024) who tested the anti-cancer activity of *Mespilus germanica* leaf extract and plant-derived *Micromonospora* sp*.* M2 by assessing their activity against MCF-7 and A549 cell lines (Jeong et al. 2024; Görmez et al. 2024). The cytotoxicity of amygdalin on two cell lines, MCF-7 and A549, revealed good anti-cancer activity. MTT assay confirmed that the IC50 values were 55.93 ± 2.16 and 54.73 ± 1.67 μg/mL for MCF-7 and A549, respectively. The observed cytotoxic activity aligns with the findings of multiple studies advocating amygdalin’s use in treating various malignant diseases, including cancers of the lung, breast, prostate, colorectal, cervical, and gastrointestinal systems. Research has demonstrated that amygdalin induces apoptosis in cancer cells, thereby inhibiting tumor growth and reducing the rate of metastatic spread (Chang et al. 2006; Chen et al. 2013; Cecarini et al. 2022; Spanoudaki et al. 2023). The current study tested the anti-cancer ability of *L. rhamnosus* as a natural agent derived from known friendly bacteria. The cytotoxicity of CFS from *L. rhamnosus* on two cancerous cell lines, MCF-7 and A549, revealed good anti-cancer activity. MTT assay demonstrated that the IC50 values were 354 ± 5.05 and 293 ± 3.09 μg/mL for MCF-7 and A549, respectively. The newly isolated *Lactobacillus hilgardii* was found to have anti-cancer and apoptotic capabilities against the Caco-2 cell line (Pourramezan et al. 2020). Another freshly isolated strain of *L. acidophilus* was proven to induce apoptosis in cancer cells, suggesting its anti-cancer activity (Nami et al. 2014).

However, combination therapy is a therapeutic approach that leverages the synergistic or additive interactions between two or more drugs. This synergy often results in enhanced treatment efficacy compared to individual drugs alone (Milczarek et al. 2021). By administering both compounds at low doses, combination therapy can reduce toxicity while targeting multiple molecular pathways. This multi-target approach can also increase drug sensitivity and improve treatment outcomes (Chen et al. 2020). Conventional chemotherapy often involves the simultaneous use of multiple drugs to treat various cancers. However, this combination therapy can lead to significant side effects. Introducing bioactive natural compounds alongside chemotherapy may enhance treatment efficacy while reducing toxic adverse effects (Chamberlin et al. 2019). Patients undergoing side effects associated with chemotherapy, such as FOLFOX chemotherapy, the most common regimen for colorectal cancer (CRC), often experience a range of adverse effects. These include gastrointestinal symptoms, neurological issues, respiratory problems, and skin conditions like hair loss (Salehifar et al. 2019). Another common chemotherapy combination, DCF (docetaxel + cisplatin + 5-FU), is associated with high toxicity. Patients may suffer from stomatitis, diarrhea, nausea, vomiting, and neuropathy (Chen et al. 2018).

Natural bioactive compounds such as amygdalin and CFS of *L. rhamnosus* offer several advantages over synthetic drugs. We found that both amygdalin and CFS of *L. rhamnosus* inhibit cancer cell growth dose-dependently. Also, their structural diversity allows them to target previously “undruggable” targets. These compounds often naturally target biologically relevant pathways due to their origins as secondary metabolites or signaling molecules. The limited overlap between the molecular targets of synthetic drugs and natural products suggests the potential for novel therapeutic approaches to cancer treatment. Moreover, leveraging existing natural compounds can reduce development costs and provide alternative options to chemotherapy (Chamberlin et al. 2019; Gouda et al. 2024). Our study examined the combined effect of amygdalin and CFS of *L. rhamnosus* on MCF-7 and A549 cancerous cells. Interaction analysis revealed a synergistic effect between amygdalin and CFS of *L. rhamnosus* on MCF-7 and A549 cell growth. This combination inhibited MCF-7 cell growth more effectively than monotherapy. It led to a significant reduction in the IC50 of amygdalin and CFS of *L. rhamnosus* from 55.93 ± 2.16 and 354 ± 5.05 µg/mL to 24.86 ± 0.185 and 131.5 ± 0.568 µg/mL, respectively. The combination therapy also reduced the IC50 of amygdalin and CFS of *L. rhamnosus* that inhibited A549 cell growth from 54.73 ± 1.67 and 293 ± 3.09 µg/mL to 17.95 ± 0.484 and 92.09 ± 0.821 μg/mL, respectively. This reduction in the IC50 suggests that it could decrease monotherapy’s toxicity and side effects.

The National Cancer Institute defines cytotoxicity levels based on IC50 values. Compounds with IC50 values below 20 µg/mL are classified as highly cytotoxic. Those with IC50 values between 21 and 200 µg/mL exhibit moderate cytotoxicity, while those between 201 and 500 µg/mL are considered weakly cytotoxic. Compounds with IC50 values exceeding 501 µg/mL are deemed non-cytotoxic (Goldin et al. 1981; Grever 1992). Consistent with these criteria, our results show that monotherapy with amygdalin and CFS of *L. rhamnosus* exhibited lower cytotoxicity in both cell lines. However, the combination of amygdalin and CFS of *Lactobacillus* demonstrated enhanced cytotoxicity against the tested cancer cell lines.

The Chou-Talalay method was used to determine the compound combination’s combination index (CI) values against MCF-7 and A549 cell lines. The CI values were 0.8159 for MCF-7 and 0.6422 for A549. A CI value below 1 indicates that combination therapy is more effective than monotherapy at inhibiting cell growth in both cell lines (Chou 2010). These results indicate a synergistic effect of the combination therapy on both cell lines, suggesting that the drugs work together more effectively than when used individually (Chou 2010). Hence, the combination of amygdalin and CFS of *L. rhamnosus* has significant potential as an in vitro anti-cancer agent.

Disrupted cell growth and programmed cell death (apoptosis) are directly linked to cancer development. Proper apoptosis, a natural mechanism that removes undesirable or damaged cells, is crucial for maintaining tissue integrity and preventing uncontrolled cell proliferation (Kroemer and Reed 2000; Khalaf et al. 2019). Consequently, therapeutic strategies targeting the apoptotic pathway hold promise in combating cancer progression. Apoptosis is characterized by morphological changes such as cell shrinkage, chromatin condensation, nuclear fragmentation, and membrane blebbing (Reed 2000). To determine if the growth inhibition in MCF-7 and A549 cell lines induced by combination therapy was due to apoptosis, we utilized PI staining and Annexin V-FITC assays. Annexin V binding, assessed through flow cytometry with dual-color staining, enables differentiation between early apoptosis, late apoptosis, and necrosis. Our findings revealed apoptotic morphological features in the treated cells. During early apoptosis, phospholipid asymmetry occurs before membrane disintegration (Vermes et al. 1995; Fadok et al. 2001). This process involves phosphatidylserine (PS) translocations from the inner to the outer leaflets of the plasma membrane. Annexin V specifically binds externalized PS in a calcium-dependent manner, while propidium iodide (PI) stains membrane-compromised cells. This combination distinguishes apoptotic (Annexin V +/PI-) cells from necrotic (Annexin V +/PI +) cells (Chen et al. 2018).

Flow cytometry was employed to quantify the extent of apoptosis in MCF-7 and A549 cell lines after treatment with single agents and their combinations. Before analysis, cells were stained with Annexin V and propidium iodide (PI). Combination therapy significantly induced apoptosis at 23.28% and 19.22% in A549 and MCF-7 cells, respectively, compared to 2.59% and 1.81% in amygdalin-treated A549 and MCF-7 cells. Treatment with CFS induced apoptosis at 26.18% and 19.51% in A549 and MCF-7, respectively, achieving levels comparable to combination therapy. However, the cytotoxicity assay demonstrated that the combination inhibited cell growth more effectively than monotherapy, significantly reducing the IC50 of amygdalin and CFS in *L. rhamnosus.* Thus, combination therapy offers the advantage of decreasing the dosage of both compounds while reducing side effects compared to monotherapy.

Notably, A549 and MCF-7 cells treated with amygdalin alone displayed higher necrosis proportions (4.32% and 9.81%, respectively) than combination therapy (1.94% and 4.43%, respectively). Chemotherapeutic agents can trigger multiple forms of cell death, including apoptosis and necrosis. Necrosis is associated with the release of cellular contents, leading to inflammation and potential disease exacerbations (Ricci and Zong 2006). Therapeutic strategies that preferentially induce apoptosis are generally more desirable. Therefore, the eco-friendly combination of amygdalin and *L. rhamnosus* CFS supports environmental sustainability and provides a clinical advantage by selectively activating apoptotic pathways while minimizing necrosis-associated complications compared to amygdalin monotherapy.

Generally, several in vivo studies and controlled clinical trials are required to determine suitable permitted doses of amygdalin, as the current use is based on the experience of practitioners. As reported in the German survey, physicians and healers use doses ranging from 3 to 9 g of amygdalin per day, consistent with former studies (Markowitsch et al. 2024). Furthermore, male rats’ testicular and liver tissues did not show toxic effects from amygdalin at doses of 50 and 100 mg/kg. However, an oxidative imbalance was observed in mice at 200 mg/kg (Albogami et al. 2020; Barakat et al. 2022).

These findings highlight a recent strategy to mitigate amygdalin-induced toxicity using a combination of amygdalin and CFS from *L. rhamnosus*. Another approach involves controlled-release mechanisms, such as HCN-releasing nano-composites, which enable the gradual release of cyanogenic compounds, thereby reducing the risk of acute cyanide toxicity (Elderdery et al. 2022; Barakat et al. 2022; Askar et al. 2023; Spanoudaki et al. 2023; Fernandes and Billa 2025).

Future studies could investigate a combination of amygdalin and *L. rhamnosus* CFS in nano formulations, which might provide dual control over cyanogenic compound release. While the nanocomposite could stabilize amygdalin and release HCN gradually, the probiotic CFS could further enhance this process by either decreasing the enzymatic degradation of cyanogenic compounds or modulating the pH to control the release rate better.

## Conclusion

Cancer remains a leading global cause of death despite significant therapeutic breakthroughs. Patient tolerance and adherence are compromised by the systemic toxicity of conventional treatments. Furthermore, all current cancer treatment modalities—including chemotherapy, surgery, radiation, immunotherapy, and targeted therapy—produce side effects that adversely affect quality of life.

The novel combination of amygdalin and *Lacticaseibacillus* CFS offers distinct therapeutic advantages such as selective induction of apoptosis in cancer cells, reduction of necrosis-related complications, and enhanced apoptotic activity. This approach also addresses environmental concerns, as it may reduce the ecological impact of traditional chemotherapeutic agents through biodegradable components, lower persistence in ecosystems, and reduced toxic by-products.

## Data Availability

The data supporting this study's findings are available upon reasonable request.
